# The case study approach

**DOI:** 10.1186/1471-2288-11-100

**Published:** 2011-06-27

**Authors:** Sarah Crowe, Kathrin Cresswell, Ann Robertson, Guro Huby, Anthony Avery, Aziz Sheikh

**Affiliations:** 1 Division of Primary Care, The University of Nottingham, Nottingham, UK; 2 Centre for Population Health Sciences, The University of Edinburgh, Edinburgh, UK; 3 School of Health in Social Science, The University of Edinburgh, Edinburgh, UK

## Abstract

The case study approach allows in-depth, multi-faceted explorations of complex issues in their real-life settings. The value of the case study approach is well recognised in the fields of business, law and policy, but somewhat less so in health services research. Based on our experiences of conducting several health-related case studies, we reflect on the different types of case study design, the specific research questions this approach can help answer, the data sources that tend to be used, and the particular advantages and disadvantages of employing this methodological approach. The paper concludes with key pointers to aid those designing and appraising proposals for conducting case study research, and a checklist to help readers assess the quality of case study reports.

## Introduction

The case study approach is particularly useful to employ when there is a need to obtain an in-depth appreciation of an issue, event or phenomenon of interest, in its natural real-life context. Our aim in writing this piece is to provide insights into when to consider employing this approach and an overview of key methodological considerations in relation to the design, planning, analysis, interpretation and reporting of case studies.

The illustrative 'grand round', 'case report' and 'case series' have a long tradition in clinical practice and research. Presenting detailed critiques, typically of one or more patients, aims to provide insights into aspects of the clinical case and, in doing so, illustrate broader lessons that may be learnt. In research, the conceptually-related case study approach can be used, for example, to describe in detail a patient's episode of care, explore professional attitudes to and experiences of a new policy initiative or service development or more generally to *'investigate contemporary phenomena within its real-life context'*[1]. Based on our experiences of conducting a range of case studies, we reflect on when to consider using this approach, discuss the key steps involved and illustrate, with examples, some of the practical challenges of attaining an in-depth understanding of a 'case' as an integrated whole. In keeping with previously published work, we acknowledge the importance of theory to underpin the design, selection, conduct and interpretation of case studies[2]. In so doing, we make passing reference to the different epistemological approaches used in case study research by key theoreticians and methodologists in this field of enquiry.

This paper is structured around the following main questions: *What is a case study? What are case studies used for? How are case studies conducted? What are the potential pitfalls and how can these be avoided? *We draw in particular on four of our own recently published examples of case studies (see Tables 1, 2, 3 and 4) and those of others to illustrate our discussion[3-7].

**Table 1:** Example of a case study investigating the reasons for differences in recruitment rates of minority ethnic people in asthma research[3]

***Context: ***Minority ethnic people experience considerably greater morbidity from asthma than the White majority population. Research has shown however that these minority ethnic populations are likely to be under-represented in research undertaken in the UK; there is comparatively less marginalisation in the US.
***Objective: ***To investigate approaches to bolster recruitment of South Asians into UK asthma studies through qualitative research with US and UK researchers, and UK community leaders.
***Study design: ***Single intrinsic case study
***The case: ***Centred on the issue of recruitment of South Asian people with asthma.
***Data collection: ***In-depth interviews were conducted with asthma researchers from the UK and US. A supplementary questionnaire was also provided to researchers.
***Analysis: ***Framework approach.
***Key findings: ***Barriers to ethnic minority recruitment were found to centre around:
1. The attitudes of the researchers' towards inclusion: The majority of UK researchers interviewed were generally supportive of the idea of recruiting ethnically diverse participants but expressed major concerns about the practicalities of achieving this; in contrast, the US researchers appeared much more committed to the policy of inclusion.
2. Stereotypes and prejudices: We found that some of the UK researchers' perceptions of ethnic minorities may have influenced their decisions on whether to approach individuals from particular ethnic groups. These stereotypes centred on issues to do with, amongst others, language barriers and lack of altruism.
3. Demographic, political and socioeconomic contexts of the two countries: Researchers suggested that the demographic profile of ethnic minorities, their political engagement and the different configuration of the health services in the UK and the US may have contributed to differential rates.
4. Above all, however, it appeared that the overriding importance of the US National Institute of Health's policy to mandate the inclusion of minority ethnic people (and women) had a major impact on shaping the attitudes and in turn the experiences of US researchers'; the absence of any similar mandate in the UK meant that UK-based researchers had not been forced to challenge their existing practices and they were hence unable to overcome any stereotypical/prejudicial attitudes through experiential learning.

**Table 2:** Example of a case study investigating the process of planning and implementing a service in Primary Care Organisations[4]

***Context: ***Health work forces globally are needing to reorganise and reconfigure in order to meet the challenges posed by the increased numbers of people living with long-term conditions in an efficient and sustainable manner. Through studying the introduction of General Practitioners with a Special Interest in respiratory disorders, this study aimed to provide insights into this important issue by focusing on community respiratory service development.
***Objective: ***To understand and compare the process of workforce change in respiratory services and the impact on patient experience (specifically in relation to the role of general practitioners with special interests) in a theoretically selected sample of Primary Care Organisations (PCOs), in order to derive models of good practice in planning and the implementation of a broad range of workforce issues.
***Study design: ***Multiple-case design of respiratory services in health regions in England and Wales.
***The cases: ***Four PCOs.
***Data collection: ***Face-to-face and telephone interviews, e-mail discussions, local documents, patient diaries, news items identified from local and national websites, national workshop.
***Analysis: ***Reading, coding and comparison progressed iteratively.
***Key findings:***
1. In the screening phase of this study (which involved semi-structured telephone interviews with the person responsible for driving the reconfiguration of respiratory services in 30 PCOs), the barriers of financial deficit, organisational uncertainty, disengaged clinicians and contradictory policies proved insurmountable for many PCOs to developing sustainable services. A key rationale for PCO re-organisation in 2006 was to strengthen their commissioning function and those of clinicians through Practice-Based Commissioning. However, the turbulence, which surrounded reorganisation was found to have the opposite desired effect.
2. Implementing workforce reconfiguration was strongly influenced by the negotiation and contest among local clinicians and managers about "ownership" of work and income.
3. Despite the intention to make the commissioning system more transparent, personal relationships based on common professional interests, past work history, friendships and collegiality, remained as key drivers for sustainable innovation in service development.
***Main limitations: ***It was only possible to undertake in-depth work in a selective number of PCOs and, even within these selected PCOs, it was not possible to interview all informants of potential interest and/or obtain all relevant documents. This work was conducted in the early stages of a major NHS reorganisation in England and Wales and thus, events are likely to have continued to evolve beyond the study period; we therefore cannot claim to have seen any of the stories through to their conclusion.

**Table 3:** Example of a case study investigating the introduction of the electronic health records[5]

***Context: ***Healthcare systems globally are moving from paper-based record systems to electronic health record systems. In 2002, the NHS in England embarked on the most ambitious and expensive IT-based transformation in healthcare in history seeking to introduce electronic health records into all hospitals in England by 2010.
***Objectives: ***To describe and evaluate the implementation and adoption of detailed electronic health records in secondary care in England and thereby provide formative feedback for local and national rollout of the NHS Care Records Service.
***Study design: ***A mixed methods, longitudinal, multi-site, socio-technical collective case study.
***The cases: ***Five NHS acute hospital and mental health Trusts that have been the focus of early implementation efforts.
***Data collection: ***Semi-structured interviews, documentary data and field notes, observations and quantitative data.
***Analysis: ***Qualitative data were analysed thematically using a socio-technical coding matrix, combined with additional themes that emerged from the data.
***Key findings:***
1. Hospital electronic health record systems have developed and been implemented far more slowly than was originally envisioned.
2. The top-down, government-led standardised approach needed to evolve to admit more variation and greater local choice for hospitals in order to support local service delivery.
3. A range of adverse consequences were associated with the centrally negotiated contracts, which excluded the hospitals in question.
4. The unrealistic, politically driven, timeline (implementation over 10 years) was found to be a major source of frustration for developers, implementers and healthcare managers and professionals alike.
***Main limitations: ***We were unable to access details of the contracts between government departments and the Local Service Providers responsible for delivering and implementing the software systems. This, in turn, made it difficult to develop a holistic understanding of some key issues impacting on the overall slow roll-out of the NHS Care Record Service. Early adopters may also have differed in important ways from NHS hospitals that planned to join the National Programme for Information Technology and implement the NHS Care Records Service at a later point in time.

**Table 4:** Example of a case study investigating the formal and informal ways students learn about patient safety[6]

***Context: ***There is a need to reduce the disease burden associated with iatrogenic harm and considering that healthcare education represents perhaps the most sustained patient safety initiative ever undertaken, it is important to develop a better appreciation of the ways in which undergraduate and newly qualified professionals receive and make sense of the education they receive.	
***Objectives: ***To investigate the formal and informal ways pre-registration students from a range of healthcare professions (medicine, nursing, physiotherapy and pharmacy) learn about patient safety in order to become safe practitioners.	
***Study design: ***Multi-site, mixed method collective case study.	
***The cases***: Eight case studies (two for each professional group) were carried out in educational provider sites considering different programmes, practice environments and models of teaching and learning.	
***Data collection and analysis: ***Structured in phases relevant to the three knowledge contexts:	
**Phase 1: Academic context**	Documentary evidence (including undergraduate curricula, handbooks and module outlines), complemented with a range of views (from course leads, tutors and students) and observations in a range of academic settings.

**Phase 2a: Organisational context**	Policy and management views of patient safety and influences on patient safety education and practice. NHS policies included, for example, implementation of the National Patient Safety Agency's *Seven Steps to Patient Safety*, which encourages organisations to develop an organisational safety culture in which staff members feel comfortable identifying dangers and reporting hazards.

**Phase 2b: Practice context**	The cultures to which students are exposed i.e. patient safety in relation to day-to-day working. NHS initiatives included, for example, a hand washing initiative or introduction of infection control measures.

***Key findings:***	
1. Practical, informal, learning opportunities were valued by students. On the whole, however, students were not exposed to nor engaged with important NHS initiatives such as risk management activities and incident reporting schemes.	
2. NHS policy appeared to have been taken seriously by course leaders. Patient safety materials were incorporated into both formal and informal curricula, albeit largely implicit rather than explicit.	
3. Resource issues and peer pressure were found to influence safe practice. Variations were also found to exist in students' experiences and the quality of the supervision available.	
***Main limitations: ***The curriculum and organisational documents collected differed between sites, which possibly reflected gatekeeper influences at each site. The recruitment of participants for focus group discussions proved difficult, so interviews or paired discussions were used as a substitute.	

## Discussion

### What is a case study?

A case study is a research approach that is used to generate an in-depth, multi-faceted understanding of a complex issue in its real-life context. It is an established research design that is used extensively in a wide variety of disciplines, particularly in the social sciences. A case study can be defined in a variety of ways (Table 5), the central tenet being the need to explore an event or phenomenon in depth and in its natural context. It is for this reason sometimes referred to as a "naturalistic" design; this is in contrast to an "experimental" design (such as a randomised controlled trial) in which the investigator seeks to exert control over and manipulate the variable(s) of interest.

**Table 5:** Definitions of a case study

Author	Definition
Stake[8]	*"A case study is both the process of learning about the case and the product of our learning" *(p.237)
Yin[1,27,28]	*"The all-encompassing feature of a case study is its intense focus on a single phenomenon within its real-life context...[Case studies are] research situations where the number of variables of interest far outstrips the number of datapoints" *(Yin 1999 p. 1211, Yin 1994 p. 13)
	*"A case study is an empirical inquiry that*
	• *Investigates a contemporary phenomenon in depth and within its real-life context, especially when*
	• *The boundaries between phenomenon and context are not clearly evident." *(Yin 2009 p18)
Miles and Huberman[23]	*"...a phenomenon of some sort occurring in a bounded context" *(p. 25)
Green and Thorogood[29]	*"In-depth study undertaken of one particular 'case', which could be a site, individual or policy" *(p. 284)
George and Bennett[12]	*"...an instance of a class of events [where] the term class of events refers to a phenomenon of scientific interest...that the investigator chooses to study with the aim of developing theory regarding causes of similarities or differences among instances (cases) of that class of events" *(p. 17)"

Stake's work has been particularly influential in defining the case study approach to scientific enquiry. He has helpfully characterised three main types of case study: *intrinsic*, *instrumental *and *collective*[8]. An *intrinsic *case study is typically undertaken to learn about a unique phenomenon. The researcher should define the uniqueness of the phenomenon, which distinguishes it from all others. In contrast, the *instrumental *case study uses a particular case (some of which may be better than others) to gain a broader appreciation of an issue or phenomenon. The *collective *case study involves studying multiple cases simultaneously or sequentially in an attempt to generate a still broader appreciation of a particular issue.

These are however not necessarily mutually exclusive categories. In the first of our examples (Table 1), we undertook an *intrinsic *case study to investigate the issue of recruitment of minority ethnic people into the specific context of asthma research studies, but it developed into a *instrumental *case study through seeking to understand the issue of recruitment of these marginalised populations more generally, generating a number of the findings that are potentially transferable to other disease contexts[3]. In contrast, the other three examples (see Tables 2, 3 and 4) employed *collective *case study designs to study the introduction of workforce reconfiguration in primary care, the implementation of electronic health records into hospitals, and to understand the ways in which healthcare students learn about patient safety considerations[4-6]. Although our study focusing on the introduction of General Practitioners with Specialist Interests (Table 2) was explicitly *collective *in design (four contrasting primary care organisations were studied), is was also *instrumental *in that this particular professional group was studied as an exemplar of the more general phenomenon of workforce redesign[4].

### What are case studies used for?

According to Yin, case studies can be used to *explain, describe *or *explore *events or phenomena in the everyday contexts in which they occur[1]. These can, for example, help to understand and explain causal links and pathways resulting from a new policy initiative or service development (see Tables 2 and 3, for example)[1]. In contrast to experimental designs, which seek to test a specific hypothesis through deliberately manipulating the environment (like, for example, in a randomised controlled trial giving a new drug to randomly selected individuals and then comparing outcomes with controls),[9] the case study approach lends itself well to capturing information on more explanatory '*how*', *'what' *and '*why*' questions, such as '*how *is the intervention being implemented and received on the ground?'. The case study approach can offer additional insights into *what *gaps exist in its delivery or *why *one implementation strategy might be chosen over another. This in turn can help develop or refine theory, as shown in our study of the teaching of patient safety in undergraduate curricula (Table 4)[6,10]. Key questions to consider when selecting the most appropriate study design are whether it is desirable or indeed possible to undertake a formal experimental investigation in which individuals and/or organisations are allocated to an intervention or control arm? Or whether the wish is to obtain a more naturalistic understanding of an issue? The former is ideally studied using a controlled experimental design, whereas the latter is more appropriately studied using a case study design.

Case studies may be approached in different ways depending on the epistemological standpoint of the researcher, that is, whether they take a critical (questioning one's own and others' assumptions), interpretivist (trying to understand individual and shared social meanings) or positivist approach (orientating towards the criteria of natural sciences, such as focusing on generalisability considerations) (Table 6). Whilst such a schema can be conceptually helpful, it may be appropriate to draw on more than one approach in any case study, particularly in the context of conducting health services research. Doolin has, for example, noted that in the context of undertaking interpretative case studies, researchers can usefully draw on a critical, reflective perspective which seeks to take into account the wider social and political environment that has shaped the case[11].

**Table 6:** Example of epistemological approaches that may be used in case study research

Approach	Characteristics	Criticisms	Key references
**Critical**	Involves questioning one's own assumptions taking into account the wider political and social environment.	It can possibly neglect other factors by focussing only on power relationships and may give the researcher a position that is too privileged.	Howcroft and Trauth[30] Blakie[31] Doolin[11,32]
	Interprets the limiting conditions in relation to power and control that are thought to influence behaviour.		Bloomfield and Best[33]
**Interpretative**	Involves understanding meanings/contexts and processes as perceived from different perspectives, trying to understand individual and shared social meanings. Focus is on theory building.	Often difficult to explain unintended consequences and for neglecting surrounding historical contexts	Stake[8] Doolin[11]
**Positivist**	Involves establishing which variables one wishes to study in advance and seeing whether they fit in with the findings. Focus is often on testing and refining theory on the basis of case study findings.	It does not take into account the role of the researcher in influencing findings.	Yin[1,27,28] Shanks and Parr[34]

### How are case studies conducted?

Here, we focus on the main stages of research activity when planning and undertaking a case study; the crucial stages are: defining the case; selecting the case(s); collecting and analysing the data; interpreting data; and reporting the findings.

#### Defining the case

Carefully formulated research question(s), informed by the existing literature and a prior appreciation of the theoretical issues and setting(s), are all important in appropriately and succinctly defining the case[8,12]. Crucially, each case should have a pre-defined boundary which clarifies the nature and time period covered by the case study (i.e. its scope, beginning and end), the relevant social group, organisation or geographical area of interest to the investigator, the types of evidence to be collected, and the priorities for data collection and analysis (see Table 7)[1]. A theory driven approach to defining the case may help generate knowledge that is potentially transferable to a range of clinical contexts and behaviours; using theory is also likely to result in a more informed appreciation of, for example, *how *and *why *interventions have succeeded or failed[13].

**Table 7:** Example of a checklist for rating a case study proposal[8]

**Communication**	Clarity: Does the proposal read well?
	Integrity: Do its pieces fit together?
	Attractiveness: Does it pique the reader's interest?
**Content**	The case: Is the case adequately defined?
	The issues: Are major research questions identified?
	Data Resource: Are sufficient data sources identified?
**Method**	Case Selection: Is the selection plan reasonable?
	Data Gathering: Are data-gathering activities outlined?
	Validation: Is the need and opportunity for triangulation indicated?
**Practicality**	Access: Are arrangements for start-up anticipated?
	Confidentiality: Is there sensitivity to the protection of people?
	Cost: Are time and resource estimates reasonable?

For example, in our evaluation of the introduction of electronic health records in English hospitals (Table 3), we defined our cases as the NHS Trusts that were receiving the new technology[5]. Our focus was on how the technology was being implemented. However, if the primary research interest had been on the social and organisational dimensions of implementation, we might have defined our case differently as a grouping of healthcare professionals (e.g. doctors and/or nurses). The precise beginning and end of the case may however prove difficult to define. Pursuing this same example, when does the process of implementation and adoption of an electronic health record system really begin or end? Such judgements will inevitably be influenced by a range of factors, including the research question, theory of interest, the scope and richness of the gathered data and the resources available to the research team.

#### Selecting the case(s)

The decision on how to select the case(s) to study is a very important one that merits some reflection. In an *intrinsic *case study, the case is selected on its own merits[8]. The case is selected not because it is representative of other cases, but because of its uniqueness, which is of genuine interest to the researchers. This was, for example, the case in our study of the recruitment of minority ethnic participants into asthma research (Table 1) as our earlier work had demonstrated the marginalisation of minority ethnic people with asthma, despite evidence of disproportionate asthma morbidity[14,15]. In another example of an *intrinsic *case study, Hellstrom et al.[16] studied an elderly married couple living with dementia to explore how dementia had impacted on *their *understanding of home, *their *everyday life and *their *relationships.

For an *instrumental *case study, selecting a "typical" case can work well[8]. In contrast to the *intrinsic *case study, the particular case which is chosen is of less importance than selecting a case that allows the researcher to investigate an issue or phenomenon. For example, in order to gain an understanding of doctors' responses to health policy initiatives, Som undertook an *instrumental *case study interviewing clinicians who had a range of responsibilities for clinical governance in one NHS acute hospital trust[17]. Sampling a "deviant" or "atypical" case may however prove even more informative, potentially enabling the researcher to identify causal processes, generate hypotheses and develop theory.

In *collective *or multiple case studies, a number of cases are carefully selected. This offers the advantage of allowing comparisons to be made across several cases and/or replication. Choosing a "typical" case may enable the findings to be generalised to theory (i.e. analytical generalisation) or to test theory by replicating the findings in a second or even a third case (i.e. replication logic)[1]. Yin suggests two or three literal replications (i.e. predicting similar results) if the theory is straightforward and five or more if the theory is more subtle. However, critics might argue that selecting 'cases' in this way is insufficiently reflexive and ill-suited to the complexities of contemporary healthcare organisations.

The selected case study site(s) should allow the research team access to the group of individuals, the organisation, the processes or whatever else constitutes the chosen unit of analysis for the study. Access is therefore a central consideration; the researcher needs to come to know the case study site(s) well and to work cooperatively with them. Selected cases need to be not only interesting but also hospitable to the inquiry [8] if they are to be informative and answer the research question(s). Case study sites may also be pre-selected for the researcher, with decisions being influenced by key stakeholders. For example, our selection of case study sites in the evaluation of the implementation and adoption of electronic health record systems (see Table 3) was heavily influenced by NHS Connecting for Health, the government agency that was responsible for overseeing the National Programme for Information Technology (NPfIT)[5]. This prominent stakeholder had already selected the NHS sites (through a competitive bidding process) to be early adopters of the electronic health record systems and had negotiated contracts that detailed the deployment timelines.

It is also important to consider in advance the likely burden and risks associated with participation for those who (or the site(s) which) comprise the case study. Of particular importance is the obligation for the researcher to think through the ethical implications of the study (e.g. the risk of inadvertently breaching anonymity or confidentiality) and to ensure that potential participants/participating sites are provided with sufficient information to make an informed choice about joining the study. The outcome of providing this information might be that the emotive burden associated with participation, or the organisational disruption associated with supporting the fieldwork, is considered so high that the individuals or sites decide against participation.

In our example of evaluating implementations of electronic health record systems, given the restricted number of early adopter sites available to us, we sought purposively to select a diverse range of implementation cases among those that were available[5]. We chose a mixture of teaching, non-teaching and Foundation Trust hospitals, and examples of each of the three electronic health record systems procured centrally by the NPfIT. At one recruited site, it quickly became apparent that access was problematic because of competing demands on that organisation. Recognising the importance of full access and co-operative working for generating rich data, the research team decided not to pursue work at that site and instead to focus on other recruited sites.

#### Collecting the data

In order to develop a thorough understanding of the case, the case study approach usually involves the collection of multiple sources of evidence, using a range of quantitative (e.g. questionnaires, audits and analysis of routinely collected healthcare data) and more commonly qualitative techniques (e.g. interviews, focus groups and observations). The use of multiple sources of data (data triangulation) has been advocated as a way of increasing the internal validity of a study (i.e. the extent to which the method is appropriate to answer the research question)[8,18-21]. An underlying assumption is that data collected in different ways should lead to similar conclusions, and approaching the same issue from different angles can help develop a holistic picture of the phenomenon (Table 2)[4].

Brazier and colleagues used a mixed-methods case study approach to investigate the impact of a cancer care programme[22]. Here, quantitative measures were collected with questionnaires before, and five months after, the start of the intervention which did not yield any statistically significant results. Qualitative interviews with patients however helped provide an insight into potentially beneficial process-related aspects of the programme, such as greater, perceived patient involvement in care. The authors reported how this case study approach provided a number of contextual factors likely to influence the effectiveness of the intervention and which were not likely to have been obtained from quantitative methods alone.

In *collective *or multiple case studies, data collection needs to be flexible enough to allow a detailed description of each individual case to be developed (e.g. the nature of different cancer care programmes), before considering the emerging similarities and differences in cross-case comparisons (e.g. to explore why one programme is more effective than another). It is important that data sources from different cases are, where possible, broadly comparable for this purpose even though they may vary in nature and depth.

#### Analysing, interpreting and reporting case studies

Making sense and offering a coherent interpretation of the typically disparate sources of data (whether qualitative alone or together with quantitative) is far from straightforward. Repeated reviewing and sorting of the voluminous and detail-rich data are integral to the process of analysis. In *collective *case studies, it is helpful to analyse data relating to the individual component cases first, before making comparisons across cases. Attention needs to be paid to variations within each case and, where relevant, the relationship between different causes, effects and outcomes[23]. Data will need to be organised and coded to allow the key issues, both derived from the literature and emerging from the dataset, to be easily retrieved at a later stage. An initial coding frame can help capture these issues and can be applied systematically to the whole dataset with the aid of a qualitative data analysis software package.

The Framework approach is a practical approach, comprising of five stages *(familiarisation; identifying a thematic framework; indexing; charting; mapping and interpretation)*, to managing and analysing large datasets particularly if time is limited, as was the case in our study of recruitment of South Asians into asthma research (Table 1)[3,24]. Theoretical frameworks may also play an important role in integrating different sources of data and examining emerging themes. For example, we drew on a socio-technical framework to help explain the connections between different elements - technology; people; and the organisational settings within which they worked - in our study of the introduction of electronic health record systems (Table 3)[5]. Our study of patient safety in undergraduate curricula drew on an evaluation-based approach to design and analysis, which emphasised the importance of the academic, organisational and practice contexts through which students learn (Table 4)[6].

Case study findings can have implications both for theory development and theory testing. They may establish, strengthen or weaken historical explanations of a case and, in certain circumstances, allow theoretical (as opposed to statistical) generalisation beyond the particular cases studied[12]. These theoretical lenses should not, however, constitute a strait-jacket and the cases should not be "forced to fit" the particular theoretical framework that is being employed.

When reporting findings, it is important to provide the reader with enough contextual information to understand the processes that were followed and how the conclusions were reached. In a collective case study, researchers may choose to present the findings from individual cases separately before amalgamating across cases. Care must be taken to ensure the anonymity of both case sites and individual participants (if agreed in advance) by allocating appropriate codes or withholding descriptors. In the example given in Table 3, we decided against providing detailed information on the NHS sites and individual participants in order to avoid the risk of inadvertent disclosure of identities[5,25].

### What are the potential pitfalls and how can these be avoided?

The case study approach is, as with all research, not without its limitations. When investigating the formal and informal ways undergraduate students learn about patient safety (Table 4), for example, we rapidly accumulated a large quantity of data. The volume of data, together with the time restrictions in place, impacted on the depth of analysis that was possible within the available resources. This highlights a more general point of the importance of avoiding the temptation to collect as much data as possible; adequate time also needs to be set aside for data analysis and interpretation of what are often highly complex datasets.

Case study research has sometimes been criticised for lacking scientific rigour and providing little basis for generalisation (i.e. producing findings that may be transferable to other settings)[1]. There are several ways to address these concerns, including: the use of theoretical sampling (i.e. drawing on a particular conceptual framework); respondent validation (i.e. participants checking emerging findings and the researcher's interpretation, and providing an opinion as to whether they feel these are accurate); and transparency throughout the research process (see Table 8)[8,18-21,23,26]. Transparency can be achieved by describing in detail the steps involved in case selection, data collection, the reasons for the particular methods chosen, and the researcher's background and level of involvement (i.e. being explicit about how the researcher has influenced data collection and interpretation). Seeking potential, alternative explanations, and being explicit about how interpretations and conclusions were reached, help readers to judge the trustworthiness of the case study report. Stake provides a critique checklist for a case study report (Table 9)[8].

**Table 8:** Potential pitfalls and mitigating actions when undertaking case study research

Potential pitfall	Mitigating action
Selecting/conceptualising the wrong case(s) resulting in lack of theoretical generalisations	Developing in-depth knowledge of theoretical and empirical literature, justifying choices made
Collecting large volumes of data that are not relevant to the case or too little to be of any value	Focus data collection in line with research questions, whilst being flexible and allowing different paths to be explored
Defining/bounding the case	Focus on related components (either by time and/or space), be clear what is outside the scope of the case
Lack of rigour	Triangulation, respondent validation, the use of theoretical sampling, transparency throughout the research process
Ethical issues	Anonymise appropriately as cases are often easily identifiable to insiders, informed consent of participants
Integration with theoretical framework	Allow for unexpected issues to emerge and do not force fit, test out preliminary explanations, be clear about epistemological positions in advance

**Table 9:** Stake's checklist for assessing the quality of a case study report[8]

1. Is this report easy to read?
2. Does it fit together, each sentence contributing to the whole?
3. Does this report have a conceptual structure (i.e. themes or issues)?
4. Are its issues developed in a series and scholarly way?
5. Is the case adequately defined?
6. Is there a sense of story to the presentation?
7. Is the reader provided some vicarious experience?
8. Have quotations been used effectively?
9. Are headings, figures, artefacts, appendices, indexes effectively used?
10. Was it edited well, then again with a last minute polish?
11. Has the writer made sound assertions, neither over- or under-interpreting?
12. Has adequate attention been paid to various contexts?
13. Were sufficient raw data presented?
14. Were data sources well chosen and in sufficient number?
15. Do observations and interpretations appear to have been triangulated?
16. Is the role and point of view of the researcher nicely apparent?
17. Is the nature of the intended audience apparent?
18. Is empathy shown for all sides?
19. Are personal intentions examined?
20. Does it appear individuals were put at risk?

## Conclusions

The case study approach allows, amongst other things, critical events, interventions, policy developments and programme-based service reforms to be studied in detail in a real-life context. It should therefore be considered when an experimental design is either inappropriate to answer the research questions posed or impossible to undertake. Considering the frequency with which implementations of innovations are now taking place in healthcare settings and how well the case study approach lends itself to in-depth, complex health service research, we believe this approach should be more widely considered by researchers. Though inherently challenging, the research case study can, if carefully conceptualised and thoughtfully undertaken and reported, yield powerful insights into many important aspects of health and healthcare delivery.

## Competing interests

The authors declare that they have no competing interests.

## Authors' contributions

AS conceived this article. SC, KC and AR wrote this paper with GH, AA and AS all commenting on various drafts. SC and AS are guarantors.

## Pre-publication history

The pre-publication history for this paper can be accessed here:

http://www.biomedcentral.com/1471-2288/11/100/prepub

## References

[B1] Yin RK Case study research, design and method 2009 4 London: Sage Publications Ltd.

[B2] Keen J Packwood T Qualitative research; case study evaluation BMJ 1995 311 444 446 7640596 10.1136/bmj.311.7002.444 PMC2550500

[B3] Sheikh A Halani L Bhopal R Netuveli G Partridge M Car J Facilitating the Recruitment of Minority Ethnic People into Research: Qualitative Case Study of South Asians and Asthma PLoS Med 2009 6 10 1 11 10.1371/journal.pmed.1000148 PMC2752116 19823568

[B4] Pinnock H Huby G Powell A Kielmann T Price D Williams S The process of planning, development and implementation of a General Practitioner with a Special Interest service in Primary Care Organisations in England and Wales: a comparative prospective case study Report for the National Co-ordinating Centre for NHS Service Delivery and Organisation R&D (NCCSDO) 2008 http://www.sdo.nihr.ac.uk/files/project/99-final-report.pdf15318916

[B5] Robertson A Cresswell K Takian A Petrakaki D Crowe S Cornford T Prospective evaluation of the implementation and adoption of NHS Connecting for Health's national electronic health record in secondary care in England: interim findings BMJ 2010 41 c4564 10.1136/bmj.c4564 PMC2933355 20813822

[B6] Pearson P Steven A Howe A Sheikh A Ashcroft D Smith P the Patient Safety Education Study Group Learning about patient safety: organisational context and culture in the education of healthcare professionals J Health Serv Res Policy 2010 15 4 10 10.1258/jhsrp.2009.009052 20075121

[B7] van Harten WH Casparie TF Fisscher OA The evaluation of the introduction of a quality management system: a process-oriented case study in a large rehabilitation hospital Health Policy 2002 60 1 17 37 10.1016/S0168-8510(01)00187-7 11879943

[B8] Stake RE The art of case study research 1995 London: Sage Publications Ltd.

[B9] Sheikh A Smeeth L Ashcroft R Randomised controlled trials in primary care: scope and application Br J Gen Pract 2002 52 482 746 51 12236280 PMC1314417

[B10] King G Keohane R Verba S Designing Social Inquiry 1996 Princeton: Princeton University Press

[B11] Doolin B Information technology as disciplinary technology: being critical in interpretative research on information systems Journal of Information Technology 1998 13 301 311 10.1057/jit.1998.8

[B12] George AL Bennett A Case studies and theory development in the social sciences 2005 Cambridge, MA: MIT Press

[B13] Eccles M the Improved Clinical Effectiveness through Behavioural Research Group (ICEBeRG) Designing theoretically-informed implementation interventions Implementation Science 2006 1 1 8 10.1186/1748-5908-1-1 PMC1436012 16722571

[B14] Netuveli G Hurwitz B Levy M Fletcher M Barnes G Durham SR Sheikh A Ethnic variations in UK asthma frequency, morbidity, and health-service use: a systematic review and meta-analysis Lancet 2005 365 9456 312 7 15664226 10.1016/S0140-6736(05)17785-X

[B15] Sheikh A Panesar SS Lasserson T Netuveli G Recruitment of ethnic minorities to asthma studies Thorax 2004 59 7 634 15223878 PMC1747077

[B16] Hellström I Nolan M Lundh U 'We do things together': A case study of 'couplehood' in dementia Dementia 2005 4 7 22 10.1177/1471301205049188

[B17] Som CV Nothing seems to have changed, nothing seems to be changing and perhaps nothing will change in the NHS: doctors' response to clinical governance International Journal of Public Sector Management 2005 18 463 477 10.1108/09513550510608903

[B18] Lincoln Y Guba E Naturalistic inquiry 1985 Newbury Park: Sage Publications

[B19] Barbour RS Checklists for improving rigour in qualitative research: a case of the tail wagging the dog? BMJ 2001 322 1115 1117 10.1136/bmj.322.7294.1115 11337448 PMC1120242

[B20] Mays N Pope C Qualitative research in health care: Assessing quality in qualitative research BMJ 2000 320 50 52 10.1136/bmj.320.7226.50 10617534 PMC1117321

[B21] Mason J Qualitative researching 2002 London: Sage

[B22] Brazier A Cooke K Moravan V Using Mixed Methods for Evaluating an Integrative Approach to Cancer Care: A Case Study Integr Cancer Ther 2008 7 5 17 10.1177/1534735407313395 18292590

[B23] Miles MB Huberman M Qualitative data analysis: an expanded sourcebook 1994 2 CA: Sage Publications Inc.

[B24] Pope C Ziebland S Mays N Analysing qualitative data. Qualitative research in health care BMJ 2000 320 114 116 10.1136/bmj.320.7227.114 10625273 PMC1117368

[B25] Cresswell KM Worth A Sheikh A Actor-Network Theory and its role in understanding the implementation of information technology developments in healthcare BMC Med Inform Decis Mak 2010 10 1 67 10.1186/1472-6947-10-67 21040575 PMC2988706

[B26] Malterud K Qualitative research: standards, challenges, and guidelines Lancet 2001 358 483 488 10.1016/S0140-6736(01)05627-6 11513933

[B27] Yin R Case study research: design and methods 1994 2 Thousand Oaks, CA: Sage Publishing

[B28] Yin R Enhancing the quality of case studies in health services research Health Serv Res 1999 34 1209 1224 10591280 PMC1089060

[B29] Green J Thorogood N Qualitative methods for health research 2009 2 Los Angeles: Sage

[B30] Howcroft D Trauth E Handbook of Critical Information Systems Research, Theory and Application 2005 Cheltenham, UK: Northampton, MA, USA: Edward Elgar

[B31] Blakie N Approaches to Social Enquiry 1993 Cambridge: Polity Press

[B32] Doolin B Power and resistance in the implementation of a medical management information system Info Systems J 2004 14 343 362 10.1111/j.1365-2575.2004.00176.x

[B33] Bloomfield BP Best A Management consultants: systems development, power and the translation of problems Sociological Review 1992 40 533 560

[B34] Shanks G Parr A Positivist, single case study research in information systems: A critical analysis Proceedings of the European Conference on Information Systems 2003 Naples

